# Determination and Pharmacokinetic Profiles of Four Active Components From *Scrophularia ningpoensis Hemsl.* in Rats

**DOI:** 10.3389/fphar.2020.612534

**Published:** 2021-01-13

**Authors:** Shunbin Luo, Lingping Xie, Jingjing Chen, Congrong Tang, Ren-ai Xu

**Affiliations:** ^1^ The People’s Hospital of Lishui, Zhejiang, China; ^2^ The First Affiliated Hospital of Wenzhou Medical University, Wenzhou, China

**Keywords:** acteoside, cinnamic acid, UPLC-MS/MS, pharmacokinetics, harpagoside, angoroside C

## Abstract

Acteoside, angoroside C, harpagoside, and cinnamic acid, which are the main bioactive ingredients of *Scrophularia ningpoensis Hemsl.*, have wide clinical use with various biological effects. A new and sensitive ultra-performance liquid chromatography-tandem mass spectrometry (UPLC-MS/MS) method was established with taxifolin as the internal standard (IS) in this study and was successfully used to study the pharmacokinetic profiles of four active components from *S. ningpoensis Hemsl.* in rats after sublingual intravenous administration. After protein precipitation with acetonitrile, the mobile phase (consisting of acetonitrile and 0.1% formic acid) was used to separate the analytes on an Acquity UPLC BEH C18 chromatography column (2.1 × 50 mm, 1.7 μm) under gradient elution. The precursor-to-product ion transitions of 623.4 → 161.3 m/z for acteoside, 783.5 → 175.0 m/z for angoroside C, 493.3 → 345.2 m/z for harpagoside and 147.2 → 103.4 m/z for cinnamic acid were monitored by mass spectrometry with negative electrospray ionization in the multiple reaction monitoring (MRM) mode. The concentration range of 10–1,000 ng/ml could be detected by this method with a lower limit of quantification (LLOQ) of 10 ng/ml for each analyte. The intra- and inter-day precision (RSD%) of the method ranged from 2.6 to 9.9% and 2.7–11.5%, respectively. Meanwhile, the accuracy (RE%) was −9.6–10.7% in this developed method. The mean recoveries of four active components from *S. ningpoensis Hemsl.* were more than 76.7% with negligible matrix effects. The four active components from *S. ningpoensis Hemsl.* were stable under multiple storage and process conditions. A new, sensitive and simple analytical method had been established and was successfully applied to the pharmacokinetic profiles of four active components from *S. ningpoensis Hemsl.* in rats after sublingual intravenous administration.

## Introduction

**FIGURE 1:**
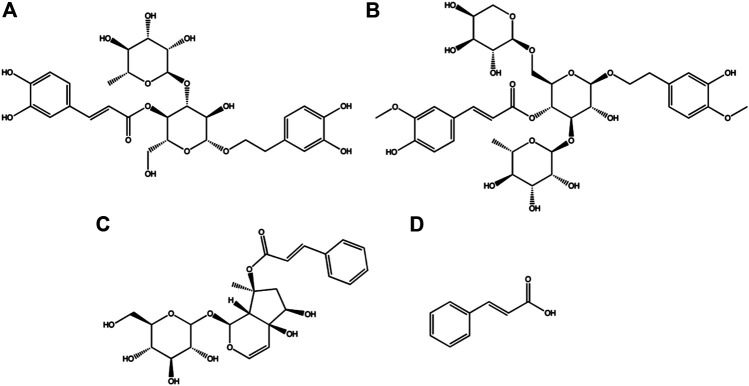
Chemical structures of acteoside **(A)**, angoroside C **(B)**, harpagoside **(C)** and cinnamic acid **(D)** in this study.

*Scrophularia ningpoensis Hemsl.* has been used in traditional Chinese medicine (TCM) for a long time (Gong et al., 2020). It is mainly used to treat diseases such as yin deficiency with fever, crimson tongue excessive thirst, maculae caused by violent heat pathogens, conjunctival congestion, pharyngalgia, scrofula, and diphtheria (Jing et al., 2011; You-Hua et al., 2014; Cao et al., 2015). *S. ningpoensis Hemsl.* has been documented in the Chinese Pharmacopoeia (2010 edition) for its ability to inhibit the proliferation of cancer cells (Kim et al., 2017). Many studies have reported a variety of biological effects from the main bioactive ingredients of *S. ningpoensis Hemsl.*, namely acteoside, angoroside C, harpagoside, and cinnamic acid (Figure 1) (Yang et al., 2009; Jing et al., 2011; Zhang et al., 2011; Cao et al., 2012; Xie et al., 2020a). Since the bioactive components determine TCM clinical effects, it is important to study the pharmacokinetics of the four active components from *S. ningpoensis Hemsl.* and to identify the mechanism of action. This identification provides an effective basis for experiments in clinical practice.

Recently, an increasing number of analytical methods have been used to characterize and determine acteoside, angoroside C, harpagoside, and cinnamic acid in biological fluids (Zhao et al., 2016; Feng et al., 2018; Zhang et al., 2018; Axmann et al., 2019; Guan et al., 2019; Zhang et al., 2019). These detection methods only determined one or two of the main bioactive ingredients of *S. ningpoensis Hemsl.*; none of the reported methods have simultaneously detected the four main active components of *S. ningpoensis Hemsl.* in biological fluids. As pharmacokinetic information is important for optimizing clinical doses, it is essential to develop a fast, simple, and sensitive analytical method for the simultaneous detection and pharmacokinetic evaluation of the various bioactive ingredients of *S. ningpoensis Hemsl.* in rat plasma.

UPLC-MS/MS has offered many advances in analytical techniques and is commonly used in environmental, bioanalytical, and pharmaceutical research (Beltifa et al., 2019; Bollen et al., 2019; Kharbouche et al., 2019; Mei et al., 2019; Xu et al., 2019; Zheng et al., 2019; Xie et al., 2020b; King et al., 2020; Wang et al., 2020). Thus, we developed a highly rugged, fast, and selective UPLC-MS/MS method to determine the concentrations of acteoside, angoroside C, harpagoside, and cinnamic acid in rat plasma. We processed 100 µL of rat plasma by protein precipitation which provided better effects and fewer matrix effects. The time savings, high sensitivity, and low matrix effects from the method were used to study the pharmacokinetics of four active components from *S. ningpoensis Hemsl.* in this work.

## Materials and Methods

### Chemicals and Reagents

Chengdu Mansite Pharmaceutical Co., Ltd. (Chengdu, China) provided the reagents, including acteoside, angoroside C, harpagoside, and cinnamic acid (purity > 98%). Taxifolin (purity > 98%), acquired from the National Institute for the Control of Pharmaceutical and Biological ProdBucts (Beijing, China), was used as an IS. Acetonitrile, methanol, and formic acid, obtained from Merck Company (Darmstadt, Germany), were of HPLC grade. The water used in this study was produced by a Milli-Q Reagent water system (Millipore, MA, United States).

### UPLC-MS/MS Conditions

The analytes were separated on an ultra-performance liquid chromatography (UPLC) unit (Waters Corp., Milford, MA, United States) through an Acquity BEH C18 column (2.1 × 50 mm, 1.7 μm particle size) and an inline 0.2 μm stainless steel frit filter (Waters Corp.). The mobile phase combining mobile phase A (acetonitrile) and mobile phase B (0.1% formic acid in water) was used under a gradient program: 0–0.4 min (10–10% A), 0.4–0.8 min (10–90% A), 0.8–2.0 min (90–90% A), and 2.0–2.1 min (90–10% A). The post time was set to 1.9 min. A 6 μL injection volume was added into the UPLC system, in which the flow rate, column temperature and autosampler temperature were 0.40 ml/min, 40°, and 10°C, respectively.

The four active components from *S. ningpoensis Hemsl.* were detected in negative ion monitoring mode by an XEVO TQD triple quadrupole mass spectrometer equipped with an electrospray ionization (ESI) source. The precursor-to-product ion transitions were m/z 623.4 → 161.3 for acteoside, m/z 783.5 → 175.0 for angoroside C, m/z 493.3 → 345.2 for harpagoside, m/z 147.2 → 103.4 for cinnamic acid and m/z 303.4 → 125.1 for IS. The instrument control and data acquisition were conducted on Masslynx 4.1 software (Waters Corp.).

### Standard Solutions, Calibration Standards and Quality Control Samples

Stock solutions (1.00 mg/ml) were diluted with methanol to prepare the standard working solutions of the *S. ningpoensis Hemsl.* active components and IS. The calibration standard solutions were diluted with drug-free plasma at the following concentrations: 10, 20, 50, 100, 200, 300, 500, and 1,000 ng/ml. The QC samples were also prepared to 20, 400, and 800 ng/ml in the same way, and IS was 200 ng/ml in acetonitrile. The prepared standard working solutions were stored in a −80°C refrigerator before analysis.

### Sample Preparation

We prepared 200 µL of IS working solution in acetonitrile (200 ng/ml) and added to a 1.5 ml centrifuge tube after 100 µL of the plasma sample was thawed to room temperature. The tubes were vortex mixed for 1.0 min and spun in a centrifuge at 13,000 × g for 10 min. The supernatant (100 µL) was collected, and 6 µL was injected into the UPLC-MS/MS system for analysis.

### Method Validation

The plasma samples, QC samples, and calibration standard solutions were run to evaluate their specificity, recovery, matrix effect, accuracy and precision, and stability (Qiu et al., 2019).

By comparing different batches of blank plasma and corresponding spiked plasma chromatograms, the specificity of the method was studied to ensure that endogenous and other substances in the sample could not interfere with the analytes and IS analysis.

The calibration curves were drawn by the peak area ratio of the analytes to IS vs. the nominal concentration of the analytes. The LLOQ was defined as the lowest concentration on the calibration curve where a signal-to-noise (S/N) was at least 10. The relative standard deviation (RSD%) and relative error (RE%) were calculated as a measure of precision and accuracy, respectively. The acceptance criteria for accuracy and precision of calibration curve data were 80–120% of the nominal concentrations and ± 20% of the nominal concentration at the LLOQ, respectively.

To assess the intra- and inter-day accuracy and precision, three different concentrations of the QC samples (20, 400, and 800 ng/ml) were detected on one day or on three consecutive days. The target RSD% and RE% were within ± 15%.

We obtained and used three different processed QC samples to evaluate the extraction recovery and matrix effect of the analytes. We compared the peak areas of samples extracted from plasma (A), postextracted blank plasma spiked samples (B), and the corresponding pure reference standard solutions (C), where A/B was defined as the extraction recovery and B/C was considered as the matrix effect.

To evaluate the stability of the analytes, we analyzed the QC samples at three different concentrations (n = 5) under different storage and process conditions (room temperature for 4 h, −80°C for 49 days, three freeze-thaw cycles from −80°C to room temperature, in the autosampler at 10°C for 48 h).

### Pharmacokinetic Study

Male Sprague-Dawley rats (180–220 g) obtained from the Laboratory Animal Center of Wenzhou Medical University (Zhejiang, China) were given free access to water and standard rat pellets. After the adaptation period, the rats were prepared for the study. We found a dose of 3.0 mg/kg of each substance in the mixture was suitable for investigating the pharmacokinetic trend in preliminary studies. All experimental procedures and protocols were performed under the National Institutes of Health Guide for the Care and Use of Laboratory Animals and were approved by the Animal Care and Use Committee of Wenzhou Medical University (wydw2018-0002) before the study began.

The caged rats were fasted for 12 h before the experiment but were allowed water. Whole blood samples (0.3 ml) collected from the tail vein were placed into the corresponding heparinized polythene tubes at the following time points after sublingual intravenous administration of mixtures containing acteoside, angoroside C, harpagoside, and cinnamic acid (3.0 mg/kg): 0.083, 0.25, 0.5, 0.75, 1, 1.5, 2, 3, 4, and 6 h. We gavaged 2 ml of saline solution to the rats 2 h after the start of the experiment to reduce animal suffering. The plasma (100 μL) was separated into a 1.5 ml Eppendorf tube after the whole blood was centrifuged at 4,000 g for 8 min. The plasma concentration vs. time data were imported into DAS 2.0 software (Drug and Statistics software, Drug Clinical Research Center, Shanghai University of Traditional Chinese Medicine, Shanghai, China) to calculate the pharmacokinetic parameters of four active components from *S. ningpoensis Hemsl*.

## Results and Discussion

### Method Development and Optimization

Sample extraction plays a key role in sensitivity and reliability and is an important factor in bioanalysis. Protein precipitation offered time-savings, simplicity, low matrix effects, and high recoveries in this research. We tried several organic solvents, including acetonitrile, methanol, and different ratios of methanol and acetonitrile, but we chose 200 µL acetonitrile as the precipitant due to its good performance.

To obtain a better chromatogram behavior and ionization effect, we tried many chromatographic columns and mobile phases with different ratios (with or without ammonium formate or formic acid). We adopted a mobile phase consisting of acetonitrile and 0.1% formic acid. When compared to other columns, such as the Waters Acquity UPLC HSS C18 column (2.1 × 100 mm, 1.8 μm), a Waters Acquity UPLC BEH C18 column (2.1 × 50 mm, 1.7 μm) with gradient elution provided better peak symmetry, appropriate analysis time, and fewer matrix effects. The whole analysis time was 4.0 min under the selected conditions.

### Method Validation

**FIGURE 2:**
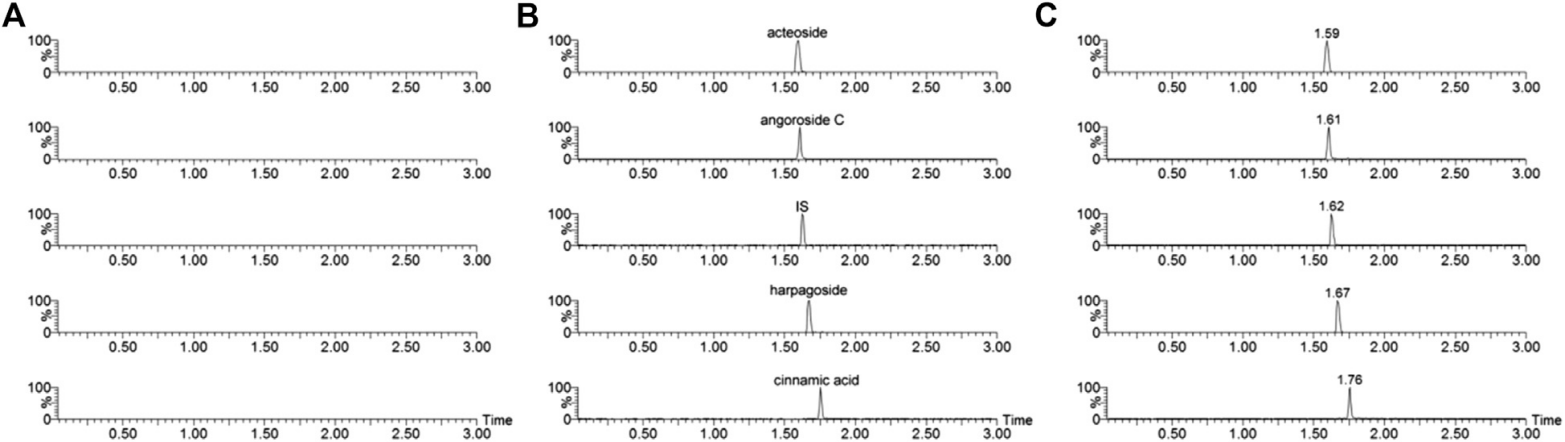
Representative chromatograms of acteoside, angoroside C, harpagoside, cinnamic acid and IS in rat plasma samples. **(A)** a blank plasma sample; **(B)** blank plasma sample spiked with acteoside, angoroside C, harpagoside, cinnamic acid and IS; **(C)** a rat plasma sample taken 0.25 h after sublingual intravenous administration of mixtures containing acteoside, angoroside C, harpagoside and cinnamic acid (3.0 mg/kg) in rats.

The representative chromatograms of the analytes and IS had no obvious interference (Figure 2). The acteoside, angoroside C, harpagoside, cinnamic acid, and IS retention times were 1.59, 1.61, 1.67, 1.76, and 1.62 min, respectively.

The method with an LLOQ of 10 ng/ml was linear over the concentration range of 10–1,000 ng/ml for the analytes. The coefficients of correlation for all the calibration curves were greater than 0.99.

The results listed in Table 1 revealed that the analyte RSDs ranged from 2.6 to 11.5%. The corresponding REs ranged from −9.6% to 10.7%, and the RSDs and REs were within the acceptable limits. The analyte recovery and matrix effect ranges were 76.7–87.1% and 92.9–110.4%, respectively.

**TABLE 1:** Precision and accuracy of acteoside, angoroside C, harpagoside and cinnamic acid in rat plasma (n = 6).

Analytes	Concentration (ng/ml)	Intra-day		Inter-day	
		RSD (%)	RE (%)	RSD (%)	RE (%)
Acteoside	20	9.9	10.3	8.5	−9.2
	400	6.1	7.2	5.3	6.6
	800	3.0	−3.5	2.7	3.1
Angoroside C	20	8.5	8.4	10.8	9.6
	400	5.8	−6.1	7.2	−6.3
	800	2.6	3.3	4.1	3.8
Harpagoside	20	9.4	−8.8	11.5	10.7
	400	6.6	−6.2	8.4	−8.3
	800	3.2	3.9	4.2	−3.4
Cinnamic acid	20	8.4	9.1	10.5	−9.6
	400	5.5	−7.2	7.7	8.2
	800	2.7	3.6	4.0	3.7

The analytes were stable according to the FDA acceptance criteria under different storage and processing conditions (room temperature for 4 h, −80°C for 49 days, three freeze-thaw cycles from −80°C to room temperature, and in the autosampler at 10°C for 48 h) (Table 2). In conclusion, the method was acceptable for studying the pharmacokinetic profiles of four active components from *S. ningpoensis Hemsl.* in rats.

**TABLE 2:** Summary of stability of QC samples under various storage conditions (n = 5).

Analytes	Concentration (ng/ml)	Stability (%, RE)			
		Short-term	Long-term	Freeze-thaw	Post-preparative
Acteoside	20	−8.4	9.1	8.8	9.7
	400	5.7	−6.7	6.0	−5.4
	800	−2.2	−3.4	2.8	3.0
Angoroside C	20	−9.6	10.8	−11.2	10.5
	400	6.1	7.9	−6.9	−7.4
	800	2.5	−4.2	3.3	−3.7
Harpagoside	20	−9.3	−11.6	10.3	9.9
	400	6.0	9.3	6.8	6.4
	800	−2.4	4.4	3.0	−3.9
Cinnamic acid	20	10.7	11.0	9.9	−10.1
	400	7.5	−8.1	−5.1	6.6
	800	3.0	3.8	−2.7	3.5

### Application of the Method in a Pharmacokinetic Study

**FIGURE 3:**
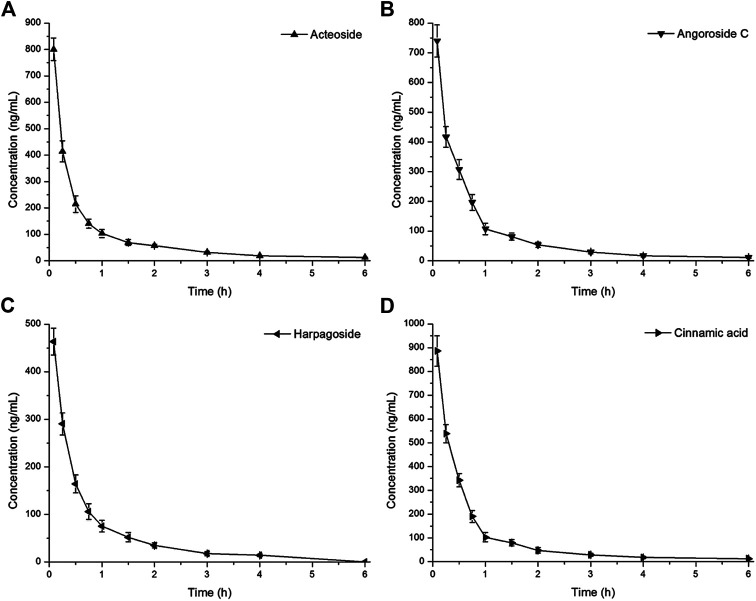
Mean plasma concentration time profiles after sublingual intravenous administration of mixtures containing acteoside **(A)**, angoroside C **(B)**, harpagoside **(C)** and cinnamic acid **(D)** (3.0 mg/kg) in six rats.

We established a UPLC-MS/MS method with taxifolin as an IS and applied it to the pharmacokinetic profile study of four active components (acteoside, angoroside C, harpagoside, and cinnamic acid) from *S. ningpoensis Hemsl.* in rats after sublingual intravenous administration of the mixture (3.0 mg/kg). The noncompartment model was used to calculate the pharmacokinetic profiles of acteoside, angoroside C, harpagoside, and cinnamic acid. The mean plasma concentration-time curves and the main pharmacokinetic parameters of the four active components are presented in Figure 3 and Table 3, respectively.

**TABLE 3:** The main pharmacokinetic parameters of the four analytes after sublingual intravenous administration to six rats.

Parameters	Acteoside	Angoroside C	Harpagoside	Cinnamic acid
t_1/2_ (h)	1.39 ± 0.23	1.57 ± 0.68	1.10 ± 0.30	1.63 ± 0.27
V_d_ (L/kg)	11.42 ± 1.92	12.38 ± 5.45	14.24 ± 3.31	11.84 ± 2.24
MRT (h)	1.28 ± 0.12	1.28 ± 0.35	1.08 ± 0.20	1.16 ± 0.15
CL (L/h/kg)	5.70 ± 0.22	5.48 ± 0.50	9.09 ± 0.84	5.04 ± 0.55
AUC_0→t_ (ng/ml·h)	511.06 ± 20.26	528.42 ± 54.45	314.65 ± 22.34	572.74 ± 67.40
AUC_0→∞_ (ng/ml·h)	527.04 ± 20.60	551.20 ± 49.52	332.28 ± 30.10	600.55 ± 70.49

Major TCM constituents are gradually metabolized and excreted after sublingual intravenous administration. The metabolism and excretion of the drug in the body are described by pharmacokinetic parameters such as clearance rate (CL), half-life (t_1/2_), and mean residence time (MRT). Among the four active components from *S. ningpoensis Hemsl.*, harpagoside had the maximum CL, but its t_1/2_, MRT, and area under the curve (AUC) were minimal. Conversely, the cinnamic acid CL was minimal, but its t_1/2_ and AUC were the maximum. The acteoside and angoriside C pharmacokinetic parameters fell between the maximum and minimum. The apparent volume of distribution (Vd) can be roughly used to infer the distribution and combination of drugs in the body. Harpagoside had the largest Vd, while acteoside had the smallest Vd. When t_1/2_ and MRT are normally short, drug metabolism and excretion are relatively fast, and Vd should be relatively small in theory. Since the complete body is a complex system, the existence of these differences needs to be confirmed by further studies.

## Conclusion

A simple, fast, and sensitive UPLC-MS/MS method was developed to simultaneously determine four active components from *S. ningpoensis Hemsl.* (acteoside, angoroside C, harpagoside and cinnamic acid) in rats after sublingual intravenous administration. This method was validated in a pharmacokinetic study. The sample was processed by acetonitrile for protein precipitation, and separation by chromatography took only 4.0 min. Clarifying the pharmacokinetic characteristics of the main TCM active ingredients is significant for accurately treating clinical diseases. It provides effective experimental basic data for clinical practice by studying the pharmacokinetics of the main bioactive components and identifying the mechanism of action. If the present study can be combined with pharmacodynamic research on the main active TCM ingredients, it can, not only avoid side effects caused by other ingredients in TCM, but can also provide scientific guidance for accurate treatment of clinical diseases with TCM.

## Data Availability Statement

The original contributions presented in the study are included in the article/Supplementary Material, further inquiries can be directed to the corresponding authors.

## Ethics Statement

The animal study was reviewed and approved by Wenzhou Medical University.

## Author Contributions

SL: Writing original draft; Conceptualization; Data curation; Formal analysis; revise; LX: Investigation; Methodology; Resources; Supervision; revise; JC: Visualization; Writing original draft; CT: Project administration; Resources; Supervision; Writing review & editing; Validation; revise; R-AX: Project administration; Resources; Software; Supervision; Writing review & editing; Validation; revise.

## Conflict of Interest

The authors declare that the research was conducted in the absence of any commercial or financial relationships that could be construed as a potential conflict of interest.
